# Neural Processing of Cognitive Control in an Emotionally Neutral Context in Anxiety Patients

**DOI:** 10.3390/brainsci11050543

**Published:** 2021-04-26

**Authors:** Nicola König, Sarah Steber, Anna Borowski, Harald R. Bliem, Sonja Rossi

**Affiliations:** 1 ICONE-Innsbruck Cognitive Neuroscience, Department for Hearing, Speech and Voice Disorders, Medical University of Innsbruck, Anichstraße 35, 6020 Innsbruck, Austria; 2 Department of Psychology, University of Innsbruck, Innrain 52f, 6020 Innsbruck, Austria

**Keywords:** event-related brain potentials (ERPs), functional near-infrared spectroscopy (fNIRS), N200, P300, flanker task, cognitive control, anxiety disorder

## Abstract

Impaired cognitive control plays a crucial role in anxiety disorders and is associated with deficient neural mechanisms in the fronto-parietal network. Usually, these deficits were found in tasks with an emotional context. The present study aimed at investigating electrophysiological and vascular signatures from event-related brain potentials (ERPs) and functional near-infrared spectroscopy (fNIRS) in anxiety patients versus healthy controls during an inhibition task integrated in an emotionally neutral context. Neural markers were acquired during the completion of a classical Eriksen flanker task. The focus of data analysis has been the ERPs N200 and P300 and fNIRS activations in addition to task performance. No behavioral or neural group differences were identified. ERP findings showed a larger N2pc and a delayed and reduced P300 for incongruent stimuli. The N2pc modulation suggests the reorienting of attention to salient stimuli, while the P300 indicates longer lasting stimulus evaluation processes due to increased task difficulty. FNIRS did not result in any significant activation potentially suggesting a contribution from deeper brain areas not measurable with fNIRS. The missing group difference in our non-emotional task indicates that no generalized cognitive control deficit but rather a more emotionally driven deficit is present in anxiety patients.

## 1. Introduction

Even though anxiety disorders are the most common psychological disorders worldwide [1], we still do not fully understand their underlying principles and neural characteristics. By deepening this knowledge, it may be possible to further optimize anxiety treatments to promote a faster and more sustainable improvement of anxiety symptoms. This in turn could relieve health care systems and economies around the world from the distinct burden of high anxiety rates [2,3,4].

### 1.1. Cognitive Control Impairments in Anxiety

One key aspect of anxiety is the inherent feeling of loss of control, or the inability to inhibit the emotional valence of certain threatening stimuli or unwanted thoughts accompanied by impaired concentration. In Generalized Anxiety Disorder (GAD), for example, uncontrollability of worry as an impaired cognitive inhibition function is related to increased disorder severity, comorbidity, and other negative outcomes [5,6]. Thus, cognitive control plays an important role in anxiety disorders and is one crucial cognitive function, which may be positively altered through therapeutic interventions (e.g., [7]). Particularly inhibition is suggested as a promising target for neurocognitive interventions [6].

Cognitive control, also referred to as executive control or attentional control, represents a conglomerate of related albeit distinct processes, including functions such as salience detection, monitoring, attention, working memory, shifting or switching, and inhibition [8,9]. Altogether, these functions are responsible for guiding attention and promoting goal-directed activity. Cognitive control is, depending on the specific target constructs, typically assessed with neuropsychological tests like the Stroop Task (e.g., [10]), the Go/No Go Task (e.g., [11]), or the Eriksen flanker task (e.g., [12]). These tasks deliver behavioral measures like error rates and response times. Outcomes are observed to underlie the “compatibility effect” [13] in a way that response time is slowed, and error rates are higher on incompatible compared to compatible trials. The general task performance in terms of response times and error rates was found to be often but not always impaired in anxiety patients [14,15,16].

### 1.2. Neural Mechanisms of Cognitive Control

Apart from behavioral measures like task performance anxiety can be detected with a variety of psycho-physiological parameters such as heart rate variability (HRV) or other cardiac measures, as well as galvanic skin conductance and blood pressure. These para-meters serve well to reflect situations of acute stress, e.g., when anxiety is induced, as well as to compare subjects suffering from anxiety disorders and healthy controls under resting conditions [17,18]. A meta-analysis including studies on resting-state HRV in anxiety patients compared to healthy controls, for example, found reduced HRV in anxiety patients [19]. Recently, a study using a combination of several psycho-physiological and electrophysiological parameters, found a beneficial effect of specific types of music presented following an induced stressful situation on several physiological measures [20].

To gain a deeper insight into more fundamental characteristics of anxiety disorders, however, it is reasonable to consider investigating neural mechanisms. Focusing on the underlying neural mechanisms of cognitive control and anxiety a variety of acquisition methods are used such as functional magnetic resonance imaging (fMRI), electroence-phalography (EEG), functional near-infrared spectroscopy (fNIRS), and magnetoence-phalography (MEG) (e.g., [21]). Neuroimaging methods like fNIRS or fMRI assess vascular responses through concentration changes of oxygenated and deoxygenated hemoglobin and blood oxygen level dependent (BOLD) signals, respectively. These methods provide information about specific brain areas and their activation patterns involved in cognitive control with a high spatial resolution. Results from these neuroimaging methods showed that the brain network supporting cognitive control includes predominantly fronto-parietal brain regions such as the prefrontal cortex, the anterior cingulate, the right anterior insula, the inferior and superior parietal cortices, the inferior temporal cortex, the occipital cortex, as well as the caudate and thalamus [22,23]. Inhibitory functions, being part of the cognitive control system, are associated with a ventral fronto-parietal network (VFPN), also called ventral attention network (VAN; [9]). Trait anxiety may be seen as an impaired recruitment of prefrontal areas that are critical to the active control of attention when a task at hand does not fully govern the allocation of attention [24,25]. These effects of hypoactivation in frontal areas in anxiety patients were observed in experiments with neutral stimuli in a non-emotional context [26], indicating that these cognitive control impairments do not seem to be related to emotional processes.

One frequently used task to assess cognitive control in a neutral context is the classical version of the Eriksen flanker task [27]. During this task, a row of five arrowheads pointing either to the right or the left direction is presented. Subjects are instructed to attend to the central arrowhead flanked either by arrowheads pointing in the same (i.e., congruent condition) or opposite direction (i.e., incongruent condition). Subjects’ task is to press a button indicating the direction of the central arrowhead. Neuroimaging studies investigating this task in healthy subjects found a larger activation in a prefrontal-anterior cingulate network for incongruent compared to congruent trials indicating a stronger need for inhibitory control [28,29,30].

Apart from the specific activation pattern for inhibitory processes, electrophysiolo-gical methods also bear the potential of gathering more detailed insights into the fast dynamic processing steps within the range of milliseconds. In the context of cognitive control, different electrophysiological measures have been investigated like event-related potentials (ERPs) or oscillatory dynamics, using EEG or MEG (e.g., [21]). For example, alpha activity is relevant for cognitive control, as decreased alpha activity is related to increased attentional and cognitive engagement [31]. Interestingly, group differences in alpha activity between high-trait anxious and low-trait anxious individuals were found only during a resting state condition but not during a flanker task [32]. Their findings led Ward et al. [32] to the assumption that high-trait anxious individuals might experience the same level of alertness during an inhibition task as low trait-anxious individuals, whereas they might be less alert during their resting state (reflected in higher alpha activity) compared to low-trait anxious individuals. Considering these missing oscillatory effects in the flanker task condition, we opted to focus on more basic ERP responses in the present study. By means of ERPs, we were able to investigate fast event-related electrophysiological correlates of inhibitory processes with respect to congruent and incongruent trials during a flanker task. In healthy subjects, an increased N200 amplitude (a negative-going deflection peaking around 200–400 ms post stimulus onset and maximal over fronto-central sites) for incongruent in contrast to congruent trials resembling a stronger degree of conflict was found [33,34,35,36,37]. Thus, the N200 component is assumed to reflect the activity of a conflict-monitoring mechanism [38]. The generators of the N200 component are assumed to be in the anterior cingulate cortex (e.g., [39], for a review refer to Folstein and Van Petten [34]). As this component was found in various versions of the flanker task but also in go/no-go, stop signal, and other spatial detection tasks creating conflict situations, it was also termed conflict N200 [33,38,40,41,42,43,44].

Furthermore, the N200 component seems to be affected by anxiety. For example, participants with high trait and state anxiety showed increased N200 amplitudes compared to low trait and state anxiety individuals in the go/no-go task [14]. Similarly, in a classical flanker task the N200 for incongruent stimuli was larger for participants with Generalized Anxiety Disorder (GAD) than healthy controls [16]. However, Cavanagh et al. [16] did not find the N200 to be a significant predictor accounting for the variance in GAD status. An ERP study [45] comparing high versus low test anxiety subjects with an emotional and a non-emotional Stroop task found a smaller N200 in high test anxiety subjects only for the emotional Stroop task, but not for the non-emotional one. Authors interpret this finding as a facilitated processing of negative (in this case test-related threatening) words as these subjects have altered cognitive schemata in regard.

Another relevant event-related brain potential (ERP) for cognitive control processes is the P300 component, peaking between 300 and 500 ms post-stimulus-onset. It is observed in a variety of cognitive tasks. A more frontally distributed P300 component (also termed P3a) is observed when attention has to be oriented to unexpected or significant events [46,47,48,49,50,51,52,53,54]. Such a P3a was also found in response inhibition tasks [35] and usually exhibits a larger amplitude on no-go trials compared to go-trials [55] and with enhanced response conflict [43]. On the other hand, a more centro-parietally distributed P300 (also termed P3b) was found in association with updating of working memory in tasks requiring attention on a target stimulus [51,56]. Moreover, the P3b is suggested to be associated with cognitive attentional processes for stimulus evaluation [57]. The parietal P300 amplitude in flanker tasks was sometimes found to be larger for congruent compared to incongruent trials which is in line with findings of various studies suggesting an amplitude reduction with increasing task difficulty [58,59,60,61].

The P300 latency was also found to be influenced by cognitive control processes. In flanker tasks, the P300 peak was later in incongruent compared to congruent trials, which was interpreted as a correlate of a stimulus evaluation mechanism [33,42,62,63,64,65,66,67].

So far, the P300 in a cognitive control context has been mostly evaluated in healthy subjects and designs which focused on the manipulation of specific factors like speed, stimulus probability/frequency, target validity, etc. (e.g., [68]). It has also been investigated in an emotional setting, e.g., by using an emotional go/no-go task which tested cognitive control in the context of emotional facial expressions [69]. In psychopathology, the P300 is a suitable parameter to investigate anxiety [70,71]. Studies analyzing the P300 component in a neutral cognitive control context in anxiety are scarce. One study investigating patients with social phobia by means of a two-tone oddball paradigm showed reductions in predominantly centroparietal P300 amplitudes and longer P300 latencies compared to healthy controls [72]. A longer P300 latency was correlated with deficits in learning processes but not executive function, measured by the Wisconsin Card Sorting Test. Klawohn et al. [73] applied an arrowhead flanker design—similar to the one used in this study—and found a reduced P300 amplitude among participants with a current depressive disorder. Thus, the P300 seems a suitable parameter for examining possible ERP differences between patients and healthy controls in a cognitive control context. To our knowledge there are no studies examining this ERP component in anxiety patients by using a classical version of the flanker task in a non-emotional context. Considering the investigation of emotional conflict in anxiety, however, subjects with generalized anxiety disorder as well as subclinical high trait anxiety performing an emotional flanker task showed lower P300 amplitudes compared to healthy low trait anxiety individuals [74].

### 1.3. Aims of the Study and Hypotheses

In the context of cognitive control, attentional processes are highly associated and interconnected with emotional processes, which becomes particularly evident in anxiety patients. Thus, some researchers argue that emotions should therefore be formalized as any other cognitive behavior (e.g., refer to [75]). The majority of cognitive control theories, however, does not view it as a distinct part of cognitive control but rather as a separate complex of emotional functions. The separation of the two concepts might therefore deliver important insights into the neural processes of anxiety disorders. Thus, this study aimed at investigating attentional and inhibitory control processes in an emotionally neutral context. In this respect, a classic version of the flanker task was used, while controlling for any potential emotional influences (e.g., subjective anxiety levels prior to measurements). Inhibition and its neural correlates were simultaneously assessed by event-related brain potentials (ERPs) extracted from electroencephalography (EEG) as well as functional near-infrared spectroscopy (fNIRS). This methodological combination is ideal as fNIRS in contrast to fMRI does not interfere with EEG signals. The advantage of these two neuroscientific methods is that EEG can excellently track fast temporal dynamics in the range of milliseconds while fNIRS allows for a solid spatial resolution of activations arising from cortical areas. In addition, behavioral parameters were assessed during the flanker task to gain a deeper understanding of the unique characteristics of anxiety disorders with respect to inhibitory control. Thus, based on the literature mentioned above the following hypotheses were defined for this study:

1. Higher task performance (higher accuracy rate, i.e., less errors as well as faster reaction times) in controls than anxiety patients is expected as anxiety is associated with distinct inhibition impairments;
2. With respect to ERPs we expect a larger N200 amplitude for incongruent compared to congruent stimuli as well as a larger P300 component for congruent compared to incongruent stimuli in healthy controls, indicating a higher degree of conflict and a larger task difficulty for incongruent stimuli;
3. Regarding the P300 component, a longer latency for incongruent compared to congruent stimuli in controls is expected reflecting more difficult stimulus evaluation processes;
4. If a general inhibition and stimulus evaluation deficit, extending beyond the emotional context, is present in anxiety patients, we expect differences between anxiety patients and controls regarding the N200 and P300 component. If, however, the deficits are restricted to emotional stimuli addressing the threatening events, a similar processing as in healthy controls will be expected;
5. Concerning fNIRS results we expect a higher activation in prefrontal areas for incongruent compared to congruent trials in healthy subjects indicating a stronger need for inhibitory control;
6. In anxiety patients a hypoactivation of frontal areas is assumed to be reflected in the fNIRS results.

So far, only few studies have investigated the N200 and P300 components as well as cortical activations within the same sample in the context of anxiety disorders. Even less studies investigated them in a non-emotional context. Moreover, the number of behavioral studies (assessing task performance measures) explicitly investigating inhibition in a non-emotional context in anxiety disorders is very small [6], which is also the case for simultaneous measurements of EEG and fNIRS in this respect.

## 2. Materials and Methods

### 2.1. Participants

**Figure 1:**
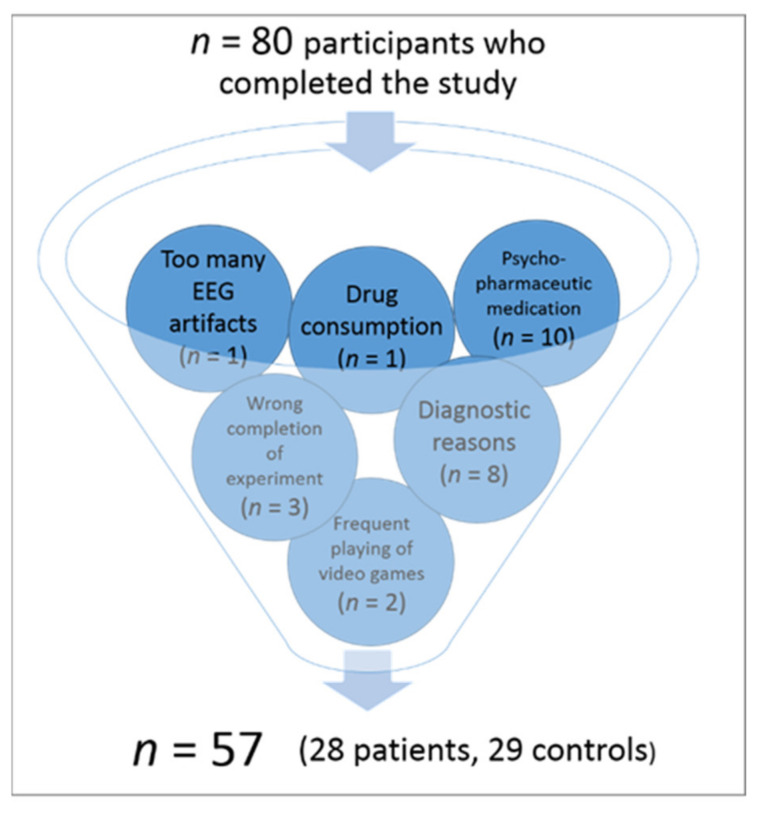
Exclusion process for selecting the EEG subjects starting with the total study sample (*n* = 80). As some participants fulfilled more than one of the exclusion criteria, the indicated n in the brackets is larger than the number of excluded participants (*n* = 23).

In total, 80 participants took part in this study. To ensure a representative sample in terms of educational levels and socio-economic status, all participants were recruited via public advertisement. At this stage, the declared inclusion criteria to take part in the study were no psychological disorder for the control group and either a professionally diagnosed anxiety disorder or subjectively elevated anxiety levels causing significant and enduring psychological distress for the patient group. General exclusion criteria communicated via email and/or phone to all participants in advance were neurological disorders, psychological disorders other than anxiety, and visual impairments. Prospective participants underwent this first exclusion process mostly during first contact by phone. This resulted in a total of 80 participants who took part in the study. A total of 23 of these subjects had to be excluded in a second exclusion process, resulting in a study sample of N = 57 participants which could be included in the final EEG analysis (for an overview of the additional exclusion factors, please refer to Figure 1). The procedure of the second exclusion process was as follows:

Before the measurement, informed consent was obtained from all participants. Then all the aforementioned exclusion and inclusion criteria were tested more specifically once again. In addition to that, more exclusion factors were identified and applied (see Figure 1). In the course of this process, 23 subjects of the original sample had to be excluded due to the following reasons: 1. diagnostic reasons (*n* = 7; e.g., the primary diagnosis was no anxiety disorder, presence of ADHD, an identified psychological disorder in the control group or only subclinical anxiety symptoms in the patient group according to the Mini DIPS interview), 2. neurological diseases (*n* = 1), 3. frequent playing of video games as potentially interfering with study outcomes due to an increased training in cognitive control abilities (*n* = 2), 4. completing the flanker task not as instructed (*n* = 3; e.g., not using index and middle finger, using both hands, etc.), 5. drug consumption (in this sample tetrahydrocannabinol; THC) less than 24 h before measurement (*n* = 1), 6. too many artifacts in the recorded EEG data (*n* = 1), and 7. intake of psychopharmaceutic medication (*n* = 10, all originally assigned to the patient group). Please note that as some participants met several exclusion criteria, the number in the brackets is larger than the total number of excluded participants (*n* = 23). Psychopharmaceutic medication reported in this sample included serotonine reuptake inhibitors (SSRIs; *n* = 9) and medication affecting the participants’ attention (methylphenidate; *n* = 1). As this medication is prescribed to enhance attentional performance, it was supposed to bias study results. Even though very common among the patient group, SSRI medication was also decided to be an exclusion factor as there are conflicting findings concerning possible effects on executive functions [76,77]. Thus, the 57 subjects finally included in EEG analyses did not take any psychopharmaceutical medication.

The remaining 57 participants (28 anxiety patients, including 24 female participants versus 29 healthy controls, including 24 female participants) were statistically analyzed. Even though gender is not equally balanced within each group but only across groups this gender distribution corresponds to the prevalence of anxiety disorders in the total population (e.g., [78]). The mean age of the analyzed sample was 25 years (range: 19–40; SD = 4.94 patient group: Mean = 26; SD = 5.70; range: 19–40; control group: Mean = 25; SD = 4.17, range: 20–38). There were no significant differences concerning age between groups (for results, please refer to Table 1).

**Figure 2:**
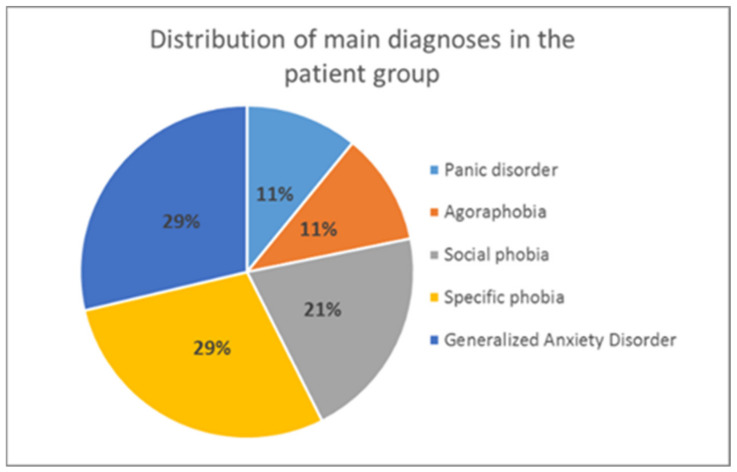
Distribution of anxiety disorders in the patient group. Only primary diagnoses are presented.

After the selection process, it was made sure that all participants in the patient group had an anxiety disorder according to the ICD-10 diagnostic criteria [79] as main diagnosis (F.40 or F.41). This was verified by the Mini DIPS interview [80] (see Section 2.2.2 for more details), which both patients and controls underwent (to detect any potential psychological disorders in the control group). To be included in the patient group, the severity of anxiety symptoms had to exceed a clinical cut-off of 4 on a scale from 1–8 (according to the Mini DIPS rating). The distribution of primary diagnoses in the analyzed patient group is presented in Figure 2.

As comorbidity with depressive disorders is very common among anxiety patients, a mild to moderate depression (assessed by the Mini DIPS interview and Beck Depression Inventory-II; [80,81,82]) was the only comorbid disorder allowed in the patient sample, together with additional, i.e., secondary anxiety symptoms and diagnoses. All other psychological disorders as well as neurological disorders were excluded from the sample (see Figure 1). Moreover, all participants had a normal or corrected-to-normal vision. A total of 54% (*n* = 15) of the patients in the analyzed sample had been in psychotherapeutic treatment any time in their life, whereas 14% (*n* = 4) of them had still been in treatment at the time of the measurement.

In addition to the diagnostic interview (Mini DIPS), all subjects underwent several psychological questionnaires (for a detailed description, please refer to Section 2.2.2). By means of that, we compared patients and controls in terms of descriptive sample characteristics and several control variables. As expected, there was a significant difference between both groups in their levels of depressive symptoms (BDI-II [82]) and their perceived quality of life (BIT [83]). According to BDI-II levels, participants in the patient group revealed a minimal current depression, whereas the control group levels fulfilled the criteria of no current depression. The two groups also differed in their perceived anxiety levels (simple Numeric Analogue Scale ranging from 1 = “no anxiety at all” to 10 = “the highest imaginable anxiety”) assessed directly before the experiment. However, this difference was only small as controls reported on average a value of 1.25 (out of 10), whereas patients reported on average a value of 2.56. In terms of current alertness (KSS score; based on the Karolinska Sleepiness Scale [84]) prior to the experiment no significant difference between both groups (*p* > 0.05) was found. Answers in both groups mostly ranged between 4, indicating “rather alert” (average answer of the control group = 3.93; SD = 1.676) and 5, which is “neither alert nor sleepy” (average answer of the patient group = 4.41; SD = 1.803). On average, measurements were carried out at about 10 am in the morning. The two groups did not differ in their caffeine intake prior to measurements (*p* > 0.05).

For subsequent fNIRS analyses, 4 further subjects had to be excluded due to technical problems during fNIRS acquisition (*n* = 2) and too many artifacts in the fNIRS signal (*n* = 2). Thus, 53 participants entered final fNIRS analyses (26 patients, 27 controls).

There was no compensation for participating in this study. The study was conducted according to the Declaration of Helsinki and approved by the local ethical committee of the Medical University of Innsbruck (Code: 1042/2017, 18 May 2017).

### 2.2. Materials

#### 2.2.1. Flanker Task

**Figure 3:**
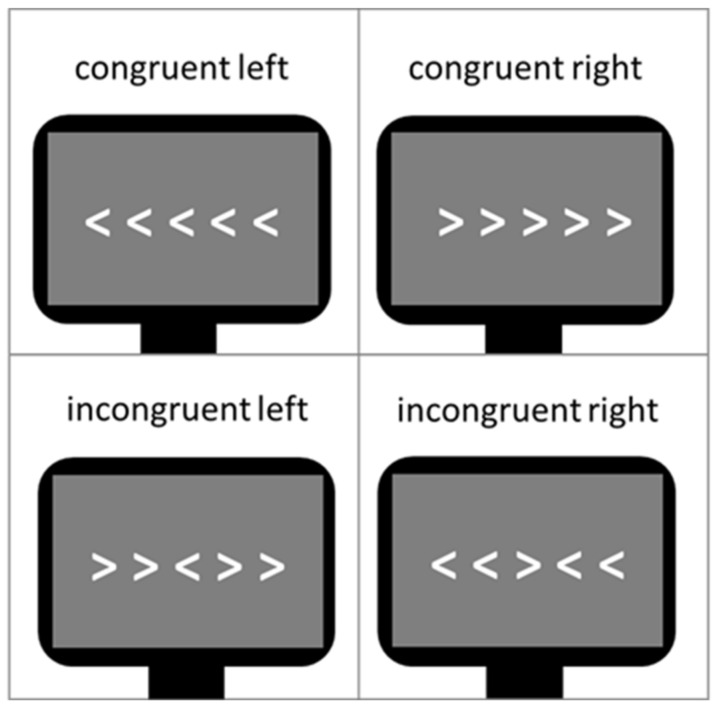
Flanker task conditions.

**Figure 4:**
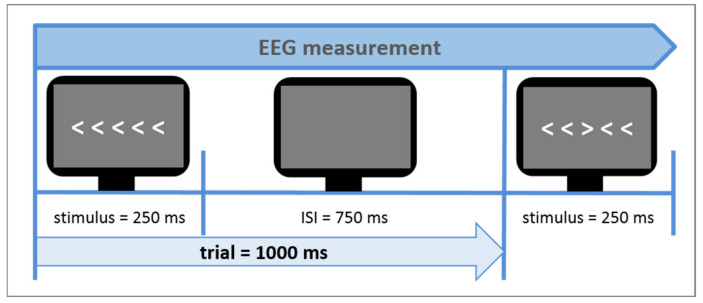
Trial sequence of the flanker task.

The Eriksen flanker task [27] was used as a measure of cognitive control during the neuroscientific experiment. Contrary to other cognitive control tasks like the Stroop Task it does not use language stimuli but non-linguistic and non-emotional visual conflict stimuli. In Figure 3, a typical example of the task is shown. The arrow version of the flanker task was also administered in this study. The central target arrowhead is flanked by non-target arrowheads, which follow the same direction (congruent flankers) or opposite direction of the target (incongruent flankers). In this study design, flankers were presented in white in the middle of a dark gray screen, serving better contrast and less eye fatigue, respectively. The size of all five arrowheads on the screen was 2 × 18 cm (0.79 × 7.1 inch). The Erikson flanker task lasted about 5 min, including 240 trials (60 congruent left direction, 60 congruent right direction, 60 incongruent left direction and 60 incongruent right direction). Since at least 30 trials per condition are needed to provide reliable measurements, a total number of 240 trials guaranteed conservative, reliable response time, and N200 amplitude measurements [85]. The participants’ response times and accuracy rates in dependence of both congruent and incongruent trials were assessed. In this study, the flanker task design was developed to be suitable for a simultaneous measurement of both EEG and functional near-infrared spectroscopy (fNIRS). The sluggish vascular response of the fNIRS, tending to overlap in time when stimuli are presented shortly one after another, thus had to be taken into consideration. Therefore, a mini-block design was introduced. It comprised 10 mini-blocks, each consisting of 24 trials with an equal distribution of right and left directed target arrowheads. Furthermore, 5 out of the 10 mini-blocks comprised a sequence of 75% congruent trials and 25% incongruent trials (i.e., mainly congruent blocks) and the other 5 mini-blocks 75% incongruent trials and 25% congruent trials (i.e., mainly incongruent blocks). Each trial consisted of the stimulus presentation (i.e., white arrowheads) on a gray screen background (duration of 250 ms), followed by a constant inter-stimulus-interval (ISI) (duration of 750 ms; see Figure 4) in which only a gray blank screen was visible. Over the entire course of the trial (1.000 ms), participants were instructed to press one of the arrow keys on the keyboard to indicate the direction of the central target arrowhead. The trial duration was fixed, hence, participants did not automatically jump to the next trial after giving an answer. In case of multiple answers per trial, only the first one was counted. If no answer was given, a missing value was logged, not an error. After each mini-block, there was a variable longer inter-block-interval (IBI) lasting 10 s on average (range 6–14 s). This extended IBI was introduced to minimize systematic overlap of the sluggish hemodynamic response assessed by the fNIRS [86]. The mini-blocks was pseudorandomized each within itself and among each other (in their order of appearance) resulting in 4 differently pseudorandomized versions for the measurements. Pseudorandomization rules within each block included maximally three consecutive congruent or incongruent trials and maximally three left or right directed target stimuli in succession, whereas pseudorandomization rules across mini-blocks included maximally three consecutive congruent or incongruent mini-blocks. In the beginning of the experiment, there were two additional training blocks of 10 trials each, which were not analyzed.

#### 2.2.2. Behavioral Measures: Standardized Psychological Tests, Response Times, and Accuracy Rates

Before the neuroscientific assessment of the flanker task, all participants underwent the Mini DIPS interview [80] to verify their conformity to the control or patient group. The Mini DIPS is a German short structured clinical interview, which serves to diagnose the most common mental disorders requiring therapeutic intervention. As a short version of the DIPS interview it can be conducted in 30 min and is a widely used diagnostic tool in clinical practice, as well as scientific studies [87,88,89]. In the present project it was conducted by specially trained collaborators [90].

Additional psychological tests and questionnaires were used as control variables for neuroscientific measurements: Beck Depression Inventory-II (BDI-II; [81,82]) served as an assessment of participant’s depression levels and potential comorbidities. The Brief Inventory of Thriving (BIT; [83]) was used as a more general comparison of psychological well-being between both groups. The BIT is a short form of the CIT (Comprehensive Inventory of Thriving; [83]) comprising 10 of the CIT items which makes it a short screening tool for mental health. To ensure the same state of alertness in both groups before the cognitive control task, all participants completed the Karolinska Sleepiness Scale (KSS; [84]), ranging from 1 to 10 with 1 = “extremely alert” to 10 = “extremely sleepy, can’t keep awake”). Two self-developed additional items were implemented to assess the participants’ caffeine intake and possible tetrahydrocannabinol (THC) consumption less than 24 h before the experiment. They were supposed to serve as additional control variables as general drug consumption was already previously assessed in the Mini DIPS interview. The two items were designed as follows: “Did you have any coffee, energy drinks or other drinks containing caffeine before this experiment?” The possible answers only broadly categorized the subjects into three options: no consumption, less than 4 h before, less than 8 h before. The item regarding THC (“Did you recently consume tetrahydrocannabinol (THC)?”) included the options: no consumption, less than 12 h before, less than 24 h before. The given time windows were based on common assumptions about the acute psychoactive effects of these substances. While caffeine consumption was equally distributed among groups it did not serve as an exclusion criterion, contrary to THC consumption, where subjects with any consumption within the last 24 h were excluded (n = 1). Participants also reported their current subjective anxiety levels directly before the task on a simple numeric analogue scale (NAS) ranging from 0 to 10 (0 = “no anxiety at all”, 10 = “highest anxiety level imaginable”) to assess possible baseline differences between patients and controls affecting task performance. A further questionnaire included demographic data and the patients’ clinical history.

During the flanker task, response times and accuracy rates were assessed in addition to the neuroscientific data.

### 2.3. Procedure

**Figure 5:**
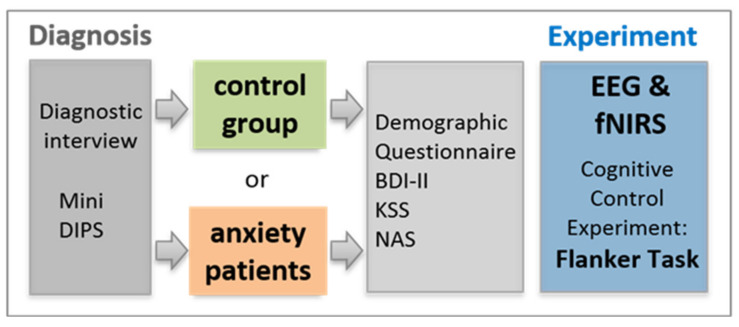
Experimental procedure.

Before the neuroscientific measurement, all subjects underwent the Mini DIPS interview to verify the correct assignment to either patient or control group. This way, patients were diagnosed or reevaluated whether a previous diagnosis still stands. Subsequently, all participants completed several paper-based standardized psychological tests and additional questionnaires (for more details, see Section 2.2.2; and for an overview, see Figure 5).

For the experimental session, the subjects were seated at an office workstation 1 m in front of a 24″ monitor. The flanker task was conducted using the Presentation software (www.neurobs.com). For the completion of the task, subjects were instructed to relax and avoid unnecessary blinking and movements, apart from indicating the direction of the central target arrowhead with the index finger or middle finger (depending on its direction) of their dominant hand. To indicate the direction of the target, participants had to press one of the corresponding arrow keys highlighted in color on a computer keyboard. The neuroscientific measurements in this project comprised a simultaneous application of electroencephalography (EEG) and functional near-infrared spectroscopy (fNIRS). For EEG, event-related brain potentials were analyzed.

### 2.4. Electroencephalography (EEG) Recordings

**Figure 6:**
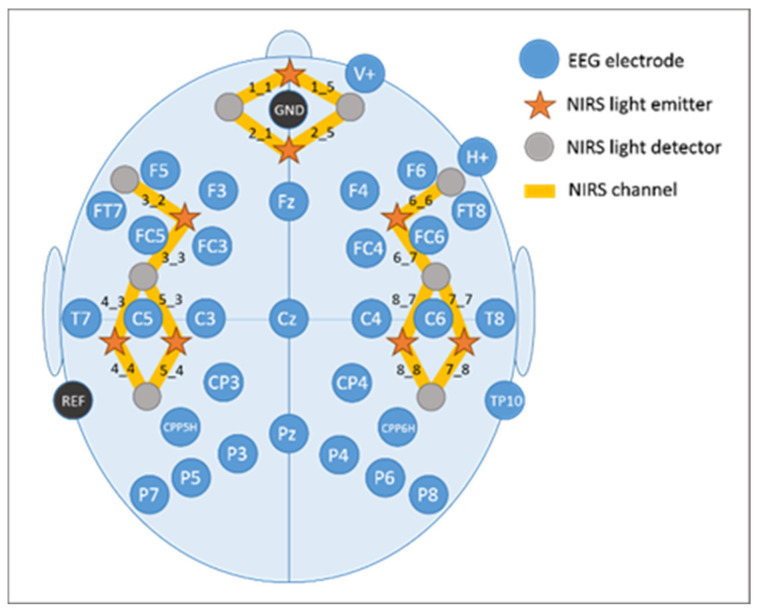
EEG electrode and fNIRS optode placement.

EEG recordings were performed with the BrainAmp System (BrainProducts GmbH, Gilching, Germany) using 32 AgAgCl active electrodes (BrainProducts GmbH, Gilching, Germany) placed according to the 10–20 placement system [91,92]. They were placed into a commercially available elastic EEG cap (EasyCap, GmbH, Herrsching, Germany) at the following positions: F5, F3, FT7, FC5, FC3, T7, C5, C3, CP3, CPP5H, P7, P5, P3, F4, F6, FC4, FC6, FT8, C4, C6, T8, CP4, CPP6H, P4, P6, P8, Fz, Pz, and Cz (Figure 6). Above the right eye and next to it, vertical and horizontal electrooculogram were recorded with electrodes FP2 and F10. One electrode served as online reference (TP9) at the left mastoid. For further re-referencing during offline analyses another electrode at the right mastoid was recorded (TP10). Position AFz was used as ground electrode. Electrode impedance was maintained below 10 kΩ (actiCAP Control, Brain Products GmbH, Gilching, Germany). The EEG signal was measured with the BrainVision Recorder (Brain Products GmbH, Gilching, Germany) software by using a sampling frequency of 1000 Hz (amplified between 0.016 and 450 Hz). Before digitalization, it was filtered by means of the analogue/digital converter with an upper cut-off of 450 Hz (24 db/oct) to prevent aliasing.

### 2.5. Functional Near-Infrared Spectroscopy (fNIRS) Recordings

Brain activation during the flanker task were measured by the functional near-infrared spectroscopy (fNIRS). This method assesses vascular changes, indexed by concentration changes of both oxygenated [oxy-Hb] and deoxygenated hemoglobin [deoxy-Hb] in cortical brain areas by means of near-infrared light penetrating the biological tissue. The physiological basis of fNIRS is the neurovascular coupling: if a brain region is more active it leads to a local increase in blood volume demanding more oxygen and glucose, which in turn leads to an increase in regional cerebral blood flow and an increase in regional blood flow velocity [93,94]. What results from these vascular and metabolic changes is a color change of the blood. The blood flow increase overcompensates oxygen consumption and elicits a focal hyperoxygenation resulting in an increase in oxygenated hemoglobin [oxy-Hb] as well as a decrease in deoxygenated hemoglobin [deoxy-Hb] [95]. [Deoxy-Hb] is inversely correlated to the BOLD signal measured by functional magnetic resonance imaging (fMRI) (for more details, see [86,96]). Calculations of concentration changes in both hemoglobins are based on a modified Beer–Lambert law [97].

The fNIRS system (NIRScout, NIRx Medizintechnik GmbH, Berlin, Germany) used in the present study sends wavelengths at 760 and 850 nm in a cw-mode. Data were recorded at a sampling rate of 7.81 Hz. The fNIRS optode arrangement included eight light emitters and eight light detectors positioned over bilateral fronto-temporo-parietal brain areas. Interoptode distance was kept at 3.5 cm. This emitter-detector configuration resulted in the assessment of 8 channels per hemisphere, covering prefrontal inferior (channels 1_1 and 1_5), prefrontal superior (channels 2_1 and 2_5), frontal (channels 3_2 and 6_6), fronto-temporal (channels 3_3 and 6_7), temporal inferior (channels 4_3 and 7_7), temporal superior (channels 5_3 and 8_7), temporo-parietal inferior (channels 4_4 and 7_8), and temporo-parietal superior (channels 5_4 and 8_8) brain regions (cf. Figure 6).

### 2.6. Data Analysis

#### 2.6.1. Behavioral Data Analyses of the Flanker Task

Comparisons between patient and control group were drawn for behavioral data in terms of task performance measures such as response times and accuracy rate during the flanker task. For each of the two behavioral parameters, a repeated measures ANOVA including the within-subject factors congruency (congruent, incongruent) and the between-subject factor group (control, patient) was computed. Post-hoc *t*-tests were calculated for all significant interactions and corrected by the False Discovery Rate (FDR) procedure [98]. Greenhouse–Geisser correction was applied whenever Mauchly’s Test of Sphericity reached significance. Significance level of *p* ≤ 0.05 was applied.

#### 2.6.2. EEG Data Analyses

Only correctly responded trials during the behavioral assessment were analyzed in the EEG. In a first step, EEG data was filtered offline with both a 50 Hz low-pass Butterworth zero-phase filter (slope: 12 dB/oct) and a Notch filter (slope: 24 dB/oct), before segmenting the data from −300 ms to 1.000 ms with 0 ms representing the time of the stimulus/flanker onset. The Gratton and Coles algorithm [99] was applied afterwards as ocular correction of vertical eye movement artifacts. Subsequently, overly contaminated channels were rejected manually and segment-wise after visual inspection of each segment for artifacts. Only the data of those subjects in whom at least 1/3 of all segments per condition in at least 15 of the 29 electrodes survived the artifact rejection process were included in the final analysis. This resulted in a cut-off of 40 trials each for the two conditions congruent and incongruent. In this process, one participant had to be excluded due to falling below the limit of minimum trials. In the next steps, a pre-stimulus baseline of 100 ms was applied and data was re-referenced to averaged mastoids (TP9, TP10). Then, event related brain potentials (ERPs) were extracted by averaging the segments for each subject and each condition.

Afterwards, a 20 ms-analysis was performed to select the optimal time windows for final statistical analysis, in addition to visual inspection of the grand averages. It was performed by means of paired-sampled *t*-tests on each electrode and separately for patients and controls between congruent and incongruent trials merged across direction of target arrowhead in consecutive 20 ms steps between 0 ms and 700 ms post-stimulus-onset. Thereby, the time windows with clusters of significant differences between congruent and incongruent trials (i.e., at least two consecutive time windows in at least two electrodes) could be identified for both groups. Results of the 20 ms-analysis revealed the following three time windows: 260–300 ms, 300–400 ms, and 420–660 ms.

For each of the identified time windows repeated measures ANOVAS on mean amplitudes including the within-subject factors congruency (congruent, incongruent) and electrode and the between-subject factor group (controls, patients) were computed. Significant interactions including at least the factor congruency (reported in Section 3.2 EEG results, Table 1) were followed up by post-hoc *t*-tests. These were adjusted with the False Discovery Rate (FDR) procedure [98] due to multiple comparisons. Greenhouse–Geisser correction [100] was applied whenever Mauchly’s Test of Sphericity reached significance. Significance level of *p* ≤ 0.05 was applied.

In order to analyze potential latency shifts, especially for the P300 component, additionally, a peak-latency analysis was performed. Amplitudes and latency values for either a negative peak (260–300 ms) or a positive peak (300–660 ms) were calculated and fed into repeated measures ANOVAS including the within-subject factors congruency (congruent, incongruent) and electrode and the between-subject factor group (controls, patients). In analogy to the mean amplitudes analysis, interactions were tested with post-hoc *t*-tests corrected with Greenhouse–Geisser [100] and FDR [98], where necessary.

#### 2.6.3. fNIRS Data Analyses

fNIRS data were analyzed mini-block-wise in order to better account for the sluggish hemodynamic response. Thus, stimulus duration was defined with 24 s (resulting from 24 trials of 250 ms presentation and 750 ms silence each) from the beginning of either a congruent or incongruent mini-block.

In order to obtain concentration changes (mmol/l) of [oxy-Hb] and [deoxy-Hb], the light reflected from the tissue was transformed by means of the modified Beer–Lambert function [97]. A manual artifact correction was performed for each participant. Artifacts (e.g., abrupt changes) were removed by a linear interpolation approach. A 0.4 Hz low pass filter (Butterworth, third order) was applied to attenuate high-frequency artefacts mainly arising from heartbeat. Next, data were correlated with a predictor generated by convolving the boxcar function of the stimulus design including both conditions congruent vs. incongruent trials with the canonical hemodynamic function [101,102] peaking at 5 s. Data were then fed into a general linear model approach to obtain beta-values for each condition as well as for each of the two hemoglobins. Statistical analyses were performed on the beta-values of both [oxy-Hb] and [deoxy-Hb]. Please note that both a decrease in [deoxy-Hb] as well as an increase in [oxy-Hb] are considered as reflections of increased activation, thus we report both hemoglobins separately [86,103].

Statistical analyses were performed separately for [oxy-Hb] and [deoxy-Hb]. For each hemoglobin, a three-way ANOVA with the within-subject factors congruency (congruent, incongruent) and fNIRS channel and the between-subject factor group (controls, patients) was performed. Significant main effects and interactions including at least the factor congruency were resolved by post-hoc *t*-tests. These were adjusted with the False Discovery Rate (FDR) procedure [98] due to multiple comparisons. Greenhouse–Geisser correction [100] was applied whenever Mauchly’s Test of Sphericity reached significance. Significance level of *p* ≤ 0.05 was applied.

## 3. Results

### 3.1. Results from Behavioral Data of the Flanker Task

The conducted repeated measures ANOVA for the response time showed a main effect of congruency (F(1,55) = 326.255, *p* < 0.0001). Having found no group differences, this main effect revealed a longer response time for incongruent trials compared to congruent ones for all participants. Mean response time for (correctly answered) congruent trials was 368.18 ms, whereas mean response time for (correctly answered) incongruent trials was 410.27 ms.

Regarding the accuracy rate (percentage of correctly answered trials) the ANOVA revealed a main effect of congruency (F(1,55) = 147.244, *p* < 0.0001) in a way that incongruent trials led to more errors (reduced accuracy) than congruent trials. The mean accuracy rate for incongruent trials was 87.35% compared to 96.84% for congruent trials.

### 3.2. EEG Results

#### 3.2.1. EEG Mean Amplitude Results

The ERP results of the repeated measures ANOVAs on mean amplitudes for all time windows are reported in Table 2 below.

##### Time Window: 300–400 ms

**Figure 7:**
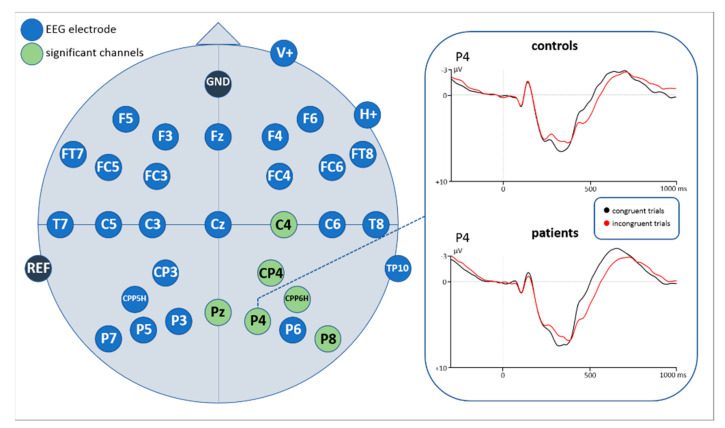
EEG results in the 260–300 ms time window, showing significant channels for both groups (patients and controls merged). The analogical curve progression of both groups is illustrated by the enlarged time course of the P4 channel. Negativity is plotted upwards.

**Figure A1:**
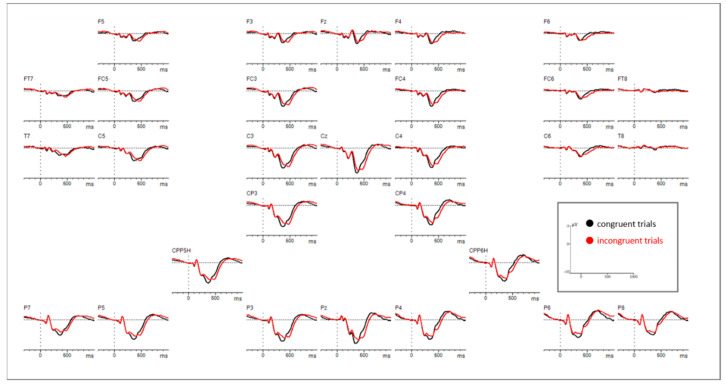
Grid view of controls (*n* = 29), showing the processing of congruent versus incongruent stimuli on all channels.

**Figure A2:**
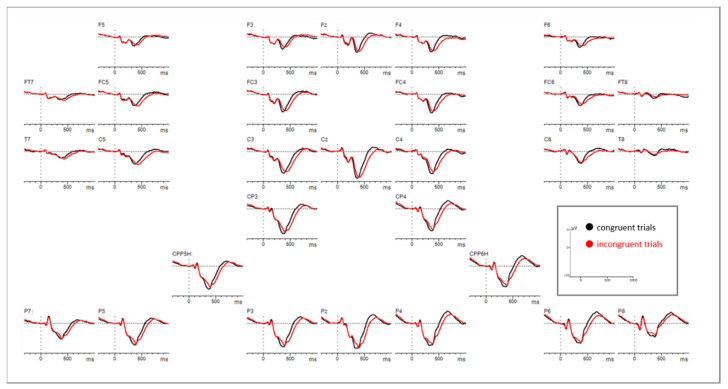
Grid view of patients (*n* = 28), showing the processing of congruent versus incongruent stimuli on all channels.

Statistical analyses of the ERP correlates with correctly answered flanker task trials for the time window 260–300 ms revealed a main effect of congruency (F(1,43) = 7.901, *p* = 0.007) as well as a significant interaction of congruency*electrode. Subsequently implemented post-hoc *t*-tests resolving the interaction revealed an increased negativity for incongruent compared to congruent flanker task trials at 6 centro-parietal and parietal electrode sites (see Figure 7, Figure A1 and Figure A2 in the Appendix B, as well as Table 3).

##### Time Window: 300–400 ms

**Figure 8:**
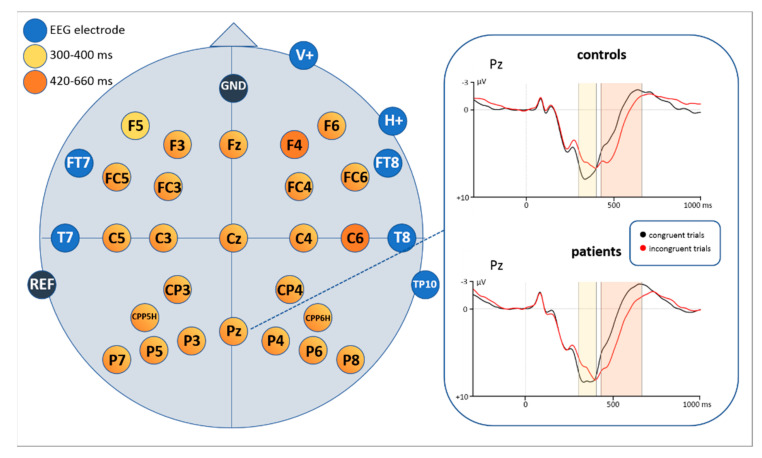
EEG results showing significant channels for the time windows 300–400 ms and 420–660 ms and their inverse effect on an exemplary channel. Negativity is plotted upwards.

Based on visual inspection of the grand averages for the time windows 300–400 ms and 420–660 ms, inverse amplitude directions between congruent and incongruent trials were identified (see Figure 8 and Figure A1 and Figure A2 in the Appendix B). Repeated measures ANOVAs were thus conducted for each time window to verify those observations.

For the 300–400 ms section a main effect of congruency (F(1,43) = 43.213, *p* < 0.0001) and a significant interaction of congruency * electrode (F(28,1204) = 10.491, *p* < 0.0001) were identified (see Table 2). Subsequently conducted post-hoc *t*-tests revealed a larger positivity for congruent trials compared to incongruent ones. Exact values for each significant EEG electrode are shown in Table A1 (Appendix A).

##### Time Window: 420–660 ms

Repeated measures ANOVA for the time window 420-660 ms after stimulus onset also revealed a main effect of congruency (F(1,43) = 39.875, *p* < 0.0001) and an interaction of congruency * electrode (F(28,1204) = 8.278, *p* < 0.0001) (see Table 2). Post-hoc *t*-tests showed more positive potentials for incongruent trials compared to congruent ones. Exact values for each significant EEG electrode are shown in Table A2 (Appendix A).

#### 3.2.2. EEG Peak-Latency Results

##### Time Window: 260–300 ms

The repeated measures ANOVA with respect to peak latencies revealed a main effect of congruency (F(1,55) = 6.418, *p* = 0.014) resulting in longer peak latencies for incongruent (277.16 ms) compared to congruent trials (274.96 ms).

The repeated measures ANOVA with respect to peak amplitudes revealed a reliable interaction congruency*electrode (F(28,1204) = 2.902, *p* = 0.012) resulting in larger negativities for incongruent than congruent trials at electrode sites CPP6H (t(56) = 2.904, *p* = 0.005) and P8 (t(55) = 3.968, *p* < 0.0001).

##### Time Window: 300–660 ms

The repeated measures ANOVA with respect to peak latencies revealed a main effect of congruency (F(1,55) = 95.335, *p* < 0.0001) resulting in longer peak latencies for incongruent (420.91 ms) compared to congruent trials (385.21 ms).

The repeated measures ANOVA with respect to peak amplitudes revealed a reliable interaction congruency*electrode (F(28,1204) = 3.113, *p* = 0.004) resulting in a larger positivity for congruent than incongruent trials at the electrode P8 (t(55) = 3.355, *p* = 0.001).

##### 3.3. fNIRS Results

Neither the repeated measures ANOVA for [oxy-Hb] nor that for [deoxy-Hb] revealed any significant main or interaction effect.

## 4. Discussion

### 4.1. Behavioral Results

Results of the behavioral data of the flanker task show that neither response times nor accuracy/error rates showed any significant group interactions or differences between patients and controls, respectively. Though contrary to our hypothesis this finding is in line with findings of other studies which found participants with anxiety disorders or high anxiety levels not to differ from controls in task performance parameters such as error rates and response times [14,16].

As expected, results showed longer response times as well as increased error rates for incongruent compared to congruent trials. This typical “compatibility effect” [13] is suggested to be an indication for increased necessity of cognitive control for incongruent trials.

In total, our initial hypothesis that controls show a higher task performance than anxiety patients (indicating distinct inhibition impairments in anxiety patients) is not corroborated by the results of this study. Possible reasons for this finding in relation to neurophysiological outcomes will be discussed in the following section.

### 4.2. N200

ERP results in this study revealed that the N200 (260–300 ms) amplitudes were significantly more negative and showed longer peak-latencies for incongruent stimuli than for congruent stimuli at several electrode sites, indicating a higher degree of conflict. A larger amplitude for incongruent stimuli is in line with previous studies [33,34,35,36], supporting the notion of the N200 to be a conflict monitoring mechanism [38]. According to our findings, the location of the channels showing this effect was over parietal and centro-parietal areas of the midline and right hemisphere. In the literature, the N200 (also referred to as conflict N200) is rather suggested to be distributed over fronto-central areas (e.g., [33]).

The question arises whether the found component might reflect an N100 component, usually found also in parietal areas as a reflection of orienting the attention to a spatial cue or task-relevant stimuli [104,105,106]. In these studies, however, the N100 component occurred within the first 200 ms after relevant stimulus-onset, which is much earlier as our negativity. A larger N100 was also found to be associated with more speeded reaction times and a better behavioral performance [107,108]. In our study, however, a larger negativity for incongruent stimuli was related to a slower reaction time and more error rates during the flanker task. Furthermore, a larger N100 was observed when more attention was required for inhibiting a button press [109]. Our study did not necessitate a response inhibition but only inhibition at the perceptual level. Due to the mentioned reasons, it seems quite unlikely that our negativity reflects an N100 component.

In a review Folstein and van Petten [34] describe several subcomponents of the N200 found in cognitive control tasks. The fronto-central conflict N200 usually elicited in flanker task studies is termed N2b. Even though this component corresponds in time with the negativity in our study, it occurs predominantly in oddball paradigms in which some stimuli were presented at a lower and others at a higher probability rate [34]. In classical flanker tasks, however, an equal proportion of congruent and incongruent stimuli is presented [41,110,111]. The same goes for this study, where an equal amount of congruent and incongruent stimuli was presented. Thus, it seems plausible to assume that our parietal N200 is not a classical fronto-central N2b.

Another subcomponent of the N200 is the so-called N2pc showing a posterior and contralateral distribution to the position of an attended object in a visual scene [112,113,114,115]. In particular, a right N2pc with a larger amplitude for incongruent compared to congruent stimuli was found in a recent flanker task presenting centered and not lateralized stimuli [116]. Authors interpret this component as reflecting reorientation of attention to salient stimuli. The right hemispheric dominance is associated to the right ventral attention network (rVAN) which is assumed the underlying neural network eliciting the right parietal N2pc [117,118,119]. The similarity in timing, distribution, and direction of effects of our negativity with the N2pc makes a similar interpretation likely. Because incongruent flanker stimuli might be more salient, they may require more attentional resources to the central arrow than congruent stimuli. Thus, the negativity in our study resembles a more attention-related ERP component rather than a pure conflict monitoring and inhibition process.

### 4.3. P300

When looking at the grand averages with respect to the P300, we first noticed that congruent stimuli were more positive than incongruent ones in an earlier time window, while a reversed direction of effects showed up in a later time window. Accordingly, we analyzed mean amplitudes in two time windows (300–400 ms and 420–660 ms). Especially in the clinical application of the P300 component its distinction between the P3a and P3b subcomponents seems to be useful [56]. They also seem to differ in their functional characteristics and recruited brain areas. According to literature, the P3a is larger in fronto-central areas associated with the activity of the anterior cingulate [56]. Functionally it is interpreted as a correlate of involuntary and transient attentional orienting mechanisms to unexpected or significant events [46,47,48,49,50,51,52] of monitoring processes [120] but it was also found as a marker of response inhibition [35]. Based on previous findings, the P3b subcomponent is more pronounced over parietal areas and assumed to be mostly related to working memory processes, next to stimulus evaluation [57,121]. Kałamała et al. [122], observed a similar inverse P300 effect in a basic flanker task comparable to our study. In their case, an earlier effect (congruent > incongruent trials; in a 370–450 ms time window) as well as a later effect (incongruent > congruent trials; in a 490–630 ms time window) were significant only at central and parietal electrode clusters while in our study the earlier and later P300 were broadly distributed over the scalp. However, pursuant to previous literature on the P300 there are also P300 latency shifts reported. Peak latencies were found to be delayed for incongruent compared to congruent stimuli, in particular in flanker tasks [35,37,123]. Such a timing pattern resembles that of reaction times for incongruent and congruent stimuli and thus suggests a reflection of stimulus evaluation time. Such an interpretation also fits with our findings. Interestingly, when performing the peak amplitude analysis for the P300 in our study, a reduced positive amplitude was attested for incongruent compared to congruent stimuli in parietal areas. A similar modulation was found in studies associating the reduction of P300 with increased task difficulty [58,59,60,61,116,124,125]. Incongruent trials in our study are more difficult to be processed as they require inhibition of incongruent flankers to correctly respond to the central arrow. Thus, more attentional resources are needed for these stimuli compared to congruent stimuli, which is not only attestable by a P300 peak amplitude and latency modulation but also behaviorally by longer reaction times for incongruent trials. Considering all performed analyses in our study, it seems plausible that our data resemble stimulus evaluation and task difficulty processes in one P300 component differing in latency, rather than in two distinct subcomponents as proposed by Kałamała [122].

### 4.4. Brain Activations Measured by fNIRS

Regarding fNIRS results we found neither differences between groups nor significant differences between congruent and incongruent trials. This might be explainable by the fact that distinguishing congruent from incongruent trials is associated with different activation levels not only in cortical frontal areas but in a broader prefrontal-anterior cingulate network [28,29,30]. Thus, it might not have been possible in our study to cover all generators of these cognitive processes with the fNIRS signal. Especially deep cortical brain areas such as the anterior cingulate (ACC), which also seems to play a role, can be measured more precisely by means of fMRI. This might be a reason for the missing difference regarding congruent versus incongruent trials in the fNIRS signal in the present study. Secondly, this outcome might be due to the specific mini-block design we used (please refer to Section 2.2.1. Flanker task for a more detailed description) to fulfil the requirements of a simultaneous EEG-fNIRS acquisition. As the mini-blocks either comprised a sequence of 75% congruent trials and 25% incongruent trials (i.e., mainly congruent blocks) or 75% incongruent trials and 25% congruent trials (i.e., mainly incongruent blocks) there might have been blurred results due to mixed conditions within mini-blocks. Consequently, existing differences between single congruent and incongruent trials might not have been detectable anymore.

### 4.5. Comparison of Anxiety Patients to Healthy Controls

Contrary to our hypotheses, no significant group differences, neither for EEG nor for fNIRS, were identified.

The missing group difference in the EEG results is in contrast to previous studies, which found differences in the N200 between patients suffering from generalized anxiety disorder and controls especially in incongruent trials (conflict N200), rather than congruent ones [16]. However, even though in a variety of disorders the N200 seems to be associated with neural deficits (mostly located in the anterior cingulate cortex (ACC)), these deficits do not always seem to result in impaired task performance [126]. Another ERP study [127] compared high versus low test anxiety subjects with an emotional and a non-emotional Stroop task. They found a smaller N200 in high test anxiety subjects only for the emotional Stroop task (including test-related threatening words), but not for the non-emotional task.

In a study by Neuhaus et al. [59] a flanker task was used in order to compare schizophrenic and depressive patients with healthy controls. They found a comparable modulation of the P300 between incongruent and congruent stimuli, with reduced P300 amplitudes for incongruent stimuli similar to our study, for both depressive patients and healthy controls. In schizophrenic patients, on the contrary, no differential P300 was attested between the two stimuli types (congruent vs. incongruent). As anxiety and depression are highly comorbid, which is also reflected in our study sample, it might be plausible to assume that no differential neural processes are present between anxiety and healthy controls when being investigated in a non-emotional context. This assumption is also supported by a study investigating the P300 component in an emotional cognitive control context: During the application of an emotional flanker task, subjects with high trait anxiety and generalized anxiety disorder showed reduced P300 amplitudes compared to healthy and low trait anxiety individuals [74]. Moreover, previous studies found the P300 to be generally modulated by affective distress, e.g., in terms of a reduced P300 during the induction of an anxious state (fear induction) compared to a neutral control condition [128]. These observations indicate that the induction of a high-fear state might disrupt attentional allocation to task relevant (but fear irrelevant) stimuli. In a study by Metzger et al. [129] higher levels of state anxiety in PTSD patients were also associated with P300 reductions. In our study, we controlled the level of state anxiety by means of a simple numeric analogue scale (NAS) just before the beginning of the experiment. Results showed a similarly relaxed state in both groups. Thus, in spite of their current anxiety disorder, anxiety patients underwent the experiment in a rather neutral and non-anxious emotional state.

Finally, a further consideration with respect to the missing ERP differentiation between anxiety patients and controls might relate to the specific ERPs we investigated (N200 and P300 components) and our focus on correctly answered trials instead of error-related ERPs such as the error-related negativity (ERN). While in our sample error rates were too small to investigate error-related EEG measures, Cavanagh et al. [16] even suggest them as being among the most promising measures for a sensitive and specific differentiation of anxiety patients and controls. According to Cavanagh et al. this may pave the way towards a new way of diagnostics by identifying a bio-signature specific to certain psychological disorders such as generalized anxiety disorder (GAD) and other anxiety disorders. In this respect, this study provides an important contribution to this endeavor by showing that error-related ERPs seem to be more beneficial measures for group differentiations than ERPs associated with correctly answered trials.

Findings from functional near-infrared spectroscopy (fNIRS) also support the notion of a rather neutral non-emotional processing in anxiety patients similar to the processing mechanisms in healthy controls. In contrast, some previous studies also including a non-emotional context and found a hypoactivation in frontal areas in anxiety patients compared to control subjects (e.g., [26]). It should be noted, however, that this study [26] used a verbal fluency task which is predominantly processed in frontal cortical brain areas. The previously mentioned argument regarding a potential contribution of deeper brain areas not accessible by fNIRS during a flanker task might also have played a role for the missing group difference in the fNIRS signal. This finding is substantiated by the fact that generators of the N200 component are assumed to be in the anterior cingulate cortex (e.g., [39], for a review refer to Folstein and Van Petten [34]).

Therefore, ERP and fNIRS findings of the present study lead to the assumption that suggested cognitive control impairments in anxiety patients, which are thought to cause their oftentimes complex anxiety symptoms, might not be detectable in an emotionally neutral context such as the classical flanker task design. The intentional absence of and control for emotional factors in our experiment and the result of missing group differences at the behavioral and neural level might support the notion that anxiety patients have no generalized deficits but rather deficits restricted to emotional and probably anxiogenic contexts. When being confronted with task irrelevant fear inducing stimuli, for example, their attentional focus might be disrupted more easily, resulting in altered task performance and neural correlates.

## 5. Conclusions

Overall, the findings of this study support the notion that anxiety patients and healthy controls equally differentiate between congruent and incongruent flanker stimuli. This was attestable at the behavioral level as both groups showed longer reaction times and more errors for incongruent trials as well as at the neural level indexed by the N2pc and the P300 in the EEG. The N2pc can be related to a reorienting of attention towards salient stimuli, which in our case were incongruent stimuli. Such a mechanism is assumed to be supported by the right ventral attention network (rVAN). The P300 in our study clearly reflects stimulus evaluation processes evidenced by longer peak latencies and task difficulty indexed by reduced P300 peak amplitudes for incongruent stimuli necessitating more attentional resources.

The fact that neither the behavioral parameters nor the neural signatures (N2pc and P300 as well as fNIRS activations) showed differences between groups suggests that anxiety patients do not manifest a generalized cognitive control deficit but only when an emotional and thus potentially anxiogenic context is present. In this classical flanker task design only one factor (inhibition) was manipulated while other factors like emotion were being controlled as much as possible leading to an almost emotionally neutral context. Therefore, our results indicate that the inhibition impairments thought to cause pathological anxiety might not only be caused by altered neural inhibition processes but also affected by emotional processes. Therefore, after all, it might not only be impaired inhibition mechanisms leading to excessive emotional reactions but also the other way round: emotional mechanisms might negatively affect cognitive control or inhibition processes.

In total, these findings contribute to a deeper understanding of the specific characteristics of neuro-cognitive processes, particularly regarding cognitive control mechanisms in anxiety patients and healthy subjects. The combination of methods served to disentangle the complex relationships between neural and behavioral variables. The results of the present study demonstrate important additional information by investigating brain–behavior relationships instead of focusing on only one of the two dimensions. For further research in this context, we suggest integrating additional physiological methods to gain a deeper understanding about the complex interdependent variables constituting anxiety disorders and cognitive control mechanisms. For example, electrocardiogram (ECG, i.e., heartbeat) recording could be a promising additional method to consider, as anxiety disorders are associated with a neuro-cardiac desynchronization due to abnormal phase-resetting [130]. Heartrate variability would also be a measure worth considering, as well as skin conductance. Integrating them would allow for a deeper insight into brain–behavior relationships and neuro-physiological mechanisms associated with anxiety. Moreover, as the data obtained by fNIRS in the present study did not reveal any significant group differences, it would be interesting to focus on slower frequency bands of the fNIRS in future research, as fMRI results have shown altered results in this frequency range when investigating healthy subjects with MRI-anxiety [131,132]. Considering the involvement of a broader brain network including deep cortical regions like the ACC in inhibition processes fNIRS might not have been a method sensitive enough to find possible differences between congruent and incongruent trials arising from these deeper regions. Therefore, conducting a similar study design using fMRI with a higher spatial resolution and coverage of signals originating from deeper brain regions might be beneficial to test this assumption.

Further research would also be helpful to clarify the possible impact of emotional aspects on impaired cognitive control mechanisms in anxiety: e.g., by investigating an emotional and a non-emotional flanker task in a within-subject design including a sample of anxiety patients compared to healthy controls. To gain more detailed insight into the characteristics of different types of anxiety disorders a design including homogenous subgroups for each anxiety disorder could be considered.

## Figures and Tables

**Table 1:** Sample description of the two intervention groups.

	Controls (*n* = 29)	Patients (*n* = 28)	*df*	*t*	*p*
Age (Mean)	24.79 (Range 20–38)	25.7 (Range 19–40)	54	−0.687	0.495
BDI-II score	4.46	13.11	53	−4.888	<0.0001
BIT	41.14	35.33	54	4.026	<0.0001
KSS score	3.93	4.41	53	−1.021	0.312
NAS	1.25	2.56	33.349	−3.794	<0.001

BDI-II = Beck Depression Inventory; BIT = Brief Inventory of Thriving; KSS = Karolinska Sleepiness Scale; NAS = Numeric Analogue Scale (ranging from 1–10).

**Table 2:** Significant ERP results of the ANOVAS with the factors congruency * electrode * group for all selected time windows.

Effect	*df*	*F*	*p*
260–300 ms			
congruency	1, 43	7.901	0.007
Congruency * electrode	28, 1204	5.724	<0.0001
300–400 ms			
Congruency	1, 43	43.213	<0.0001
Congruency * electrode	28, 1204	10.491	<0.0001
420–660 ms			
Congruency	1, 43	39.875	<0.0001
Congruency * electrode	28, 1204	8.278	<0.0001

**Table 3:** Results of post-hoc *t*-tests comparing congruency levels for time window 260–300 ms merged across both groups.

Electrode	*df*	*t*	*p*
C4	56	3.648	0.001
CP4	56	3.523	0.001
CPP6H	56	3.767	<0.0001
P4	56	3.828	<0.0001
P8	55	4.729	<0.0001
Pz	56	3.742	<0.0001

No significant differences between the two groups (patients versus controls) were found.

## Data Availability

The data presented in this study are openly available in zenodo.org at http://doi.org/10.5281/zenodo.4719209.

## Appendix A

**Table A1:** Results of post-hoc *t*-tests comparing congruency levels for time window 300–400 ms.

Electrode	*df*	*t*	*p*
F3	55	5.720	<0.0001
F5	55	2.968	0.004
FC3	56	4.771	<0.0001
FC5	56	2.819	0.007
C3	56	5.779	<0.0001
C5	56	3.207	0.002
CP3	56	5.551	<0.0001
CPP5H	56	5.551	<0.0001
P3	56	5.778	<0.0001
P5	56	5.007	<0.0001
P7	56	3.681	0.001
F6	55	2,970	0.004
FC4	56	5.471	<0.0001
FC6	56	4.298	<0.0001
C4	56	6.425	<0.0001
CP4	56	4.278	<0.0001
CPP6H	56	4.202	<0.0001
P4	56	3.670	0.001
P6	56	3.312	0.002
P8	55	4.807	<0.0001
Fz	54	4.214	<0.0001
Cz	56	4.487	<0.0001
Pz	56	5.101	<0.0001

**Table A2:** Results of post-hoc *t*-tests comparing congruency levels for time window 420–660 ms.

Electrode	*df*	*t*	*p*
F3	55	−3.313	0.002
FC3	56	−4.391	<0.0001
FC5	56	−3.170	0.002
C3	56	−5.446	<0.0001
C5	56	−3.568	0.001
CP3	56	−5.167	<0.0001
CPP5H	56	−5.634	<0.0001
P3	56	−6.355	<0.0001
P5	56	−5.676	<0.0001
P7	56	−3.919	<0.0001
F4	56	−4.086	<0.0001
F6	55	−4.747	<0.0001
FC4	56	−6.454	<0.0001
FC6	56	−5.186	<0.0001
C4	56	−7.987	<0.0001
C6	54	−4.656	<0.0001
CP4	56	−6.041	<0.0001
CPP6H	56	−5.712	<0.0001
P4	56	−5.397	<0.0001
P6	56	−3.739	<0.0001
P8	55	−4.380	<0.0001
Fz	54	−4.539	<0.0001
Cz	56	−5.957	<0.0001
Pz	56	−7.039	<0.0001
